# Case Report: Ictal hypersalivation: a stereoelectroencephalography exploration

**DOI:** 10.3389/fsurg.2025.1535408

**Published:** 2025-02-26

**Authors:** Sumika Ouchida, Armin Nikpour, David Neville Wilson, Greg Fairbrother

**Affiliations:** ^1^ Department of Neurology, Royal Prince Alfred Hospital, Sydney, NSW, Australia; ^2^ Faculty of Medicine and Health, School of Health Sciences, The University of Sydney, Camperdown, NSW, Australia; ^3^ Department of Neurosurgery, Royal Prince Alfred Hospital, Sydney, NSW, Australia; ^4^ Sydney Local Health District, Sydney, NSW, Australia; ^5^ Faculty of Medicine and Health, Susan Wakil School of Nursing and Midwifery, The University of Sydney, Sydney, NSW, Australia

**Keywords:** ictal, hypersalivation, epilepsy, stereoelectroencephalography, radiofrequency thermocoagulation

## Abstract

Epilepsy is a chronic neurological condition with various etiologies, and recurrent unprovoked seizures characterize it. Hypersalivation is a recognized symptom of insular-opercular epilepsies. A wide range of symptoms can occur during a seizure, including hypersalivation, autonomic responses, oropharyngeal sensations, visceral sensations, somatosensory disturbances, and emotional manifestations. In this case study, we examine a unique scenario of a patient who experienced predominantly salivary seizures. Hypersalivation, pill-rolling movements, and lip-smacking characterized these seizures. Importantly, the patient became seizure-free after undergoing radiofrequency thermocoagulation (RFTC) with the assistance of Stereoelectroencephalography (SEEG). Our discussion will focus on the treatment approach involving SEEG-guided RFTC and the careful identification of the brain cortex responsible for triggering excessive salivation during seizures.

## Introduction

Recurrent unprovoked seizures characterize epilepsy. Hypersalivation is an opercular syndrome, a central, Rolandic operculum ictal manifestation and other childhood epilepsy syndromes (1). A pure salivatory seizure constitutes a rare variant of focal seizure, primarily distinguished by excessive salivation as the predominant or singular symptom. This type of seizure typically arises from the insular cortex, opercular region, or adjacent brain structures. A variety of symptoms related to hypersalivation may occur during a seizure event. These symptoms can include hypersalivation, autonomic responses, sensations in the oropharynx, visceral sensations, somatosensory disturbances, and emotional responses. These manifestations can indicate different types of epilepsy syndromes (2). In cases of acquired epilepsy affecting the temporal, parietal, and frontal lobes, hypersalivation has been occasionally reported (3). Hypersalivation has been associated with temporal lobe seizures, accompanying excessive chewing and foaming at the mouth. Seizures involving the insular lobe may result in hypersalivation due to perioral and pharyngeal dystonia and increased dilute and watery saliva (2). Distinguishing between these mechanisms is important for accurately diagnosing and treating episodic salivation.

Stereo-electroencephalography (SEEG) uses invasive techniques to confirm or disprove hypotheses seeking to identify the epileptogenic zone (EZ) in patients who are suffering from drug-resistant focal epilepsy (4). Lüders et al. defined the EZ as the cortical area necessary for initiating seizures and whose removal (or disconnection) is necessary for seizure abolition (5). SEEG is used after non-invasive pre-operative evaluations. It records brain activity in 3D, precisely identifying the EZ and associated epileptic network. SEEG electrodes serve two purposes: (1) to identify the EZ by recording seizures and cortical electrical stimulation, (2) to enable radiofrequency thermocoagulation (RFTC) for treating deep-seated EZ (4). SEEG is crucial for diagnosing seizure onset in the insula due to its location in a concealed cortex region, enclosed by the frontal, parietal, and temporal opercular cortices (6). During this case discussion, we will explore the potential of SEEG-guided RFTC (SEEGg-RFTC) treatment and investigate which brain cortex induces hypersalivation during seizures.

## Case study

The patient was a 26-year-old female with a history of anxiety and depression who had been experiencing seizures since 2019. She described the seizures as starting with a tingling sensation on both sides of her mouth, followed by increased salivation and spitting. During the seizure, she would perform wringing or pill-rolling movements with both hands, with lip-smacking and mumbling. She also vomited sometimes. On two occasions, she felt heavy pressure in her chest and was taken to the hospital, where cardiac tests were unremarkable. She was initially prescribed Levetiracetam, but it caused severe depression and was ceased. She had been taking a combination of three anti-seizure medications (ASMs) for two years: Perampanel, Carbamazepine, and Zonisamide. She attempted to lose weight by going to the gym but noticed that her seizures were increasing in frequency. She was then advised against attending the gym, which increased her anxiety. The frequency of seizures restricted her independence, including her ability to drive. She expressed a loss of confidence and became emotional when discussing her condition. She also reported poor memory and a reliance on lists. She has a strong family history of epilepsy on her father's side.

From 2022 to 2024, she underwent multiple tests, including neuropsychological, magnetic resonance imaging (MRI), fluorodeoxyglucose (FDG)-positron emission tomography (PET), computed tomography (CT), five-day video-electroencephalography (VEEG) monitoring, and ten-day SEEG monitoring. During SEEG monitoring in 2024, she took Perampanel 4 mg at night, Lacosamide 200 mg twice daily, Carbamazepine 300 mg twice daily, Zonisamide 100 mg in the morning, and 200 mg at night.

**Figure 1:**
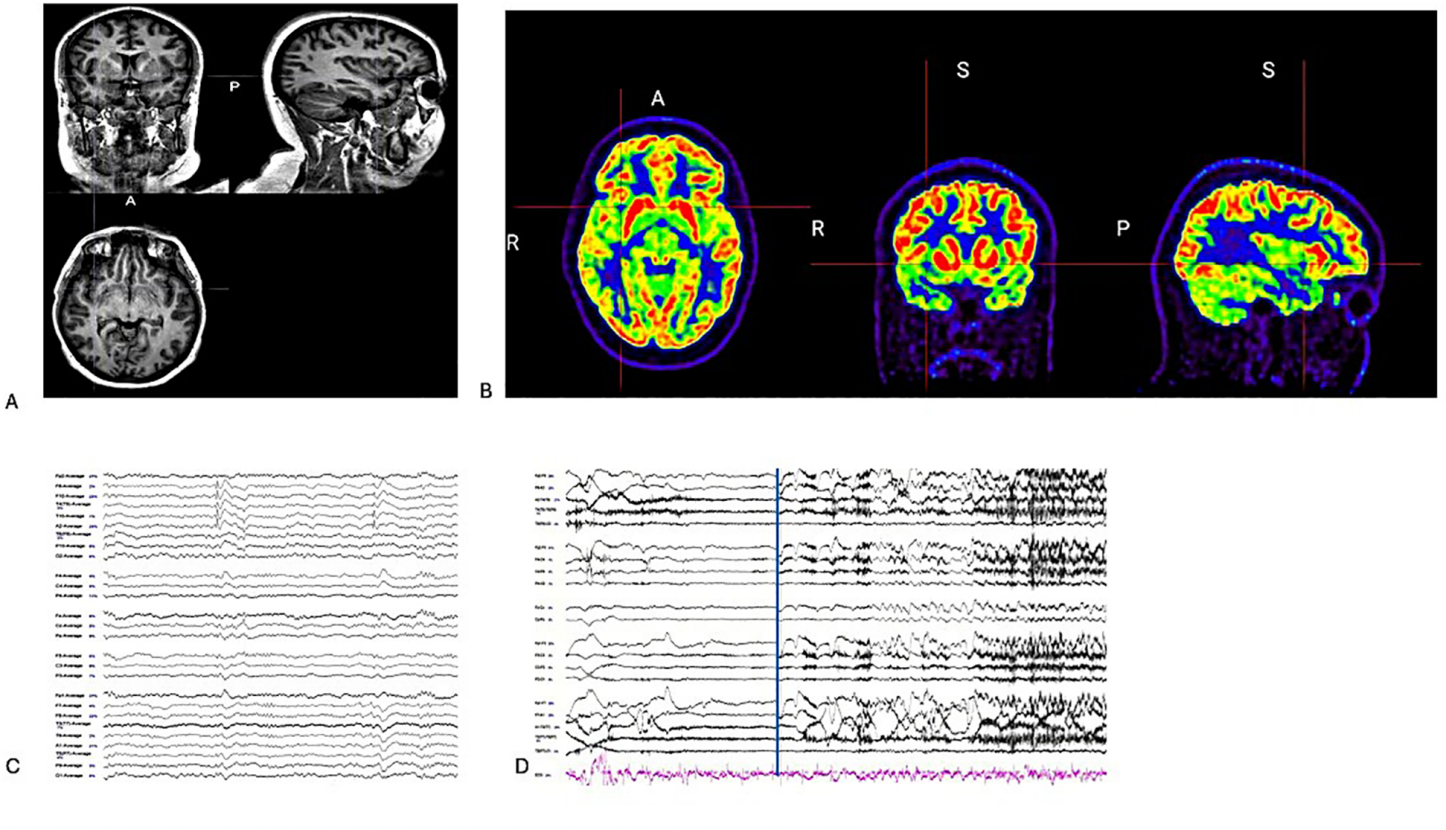
**(A)** Coronal/sagittal and axial T1 weight MRI. **(B)** Coronal/Sagittal and Axial PET-co-registered MRI showed hypometabolism in the right insula cortex. **(C)** On average montage, there are interictal discharges in the right temporal region during sleep stage 2. **(D)** On bipolar montage, the EEG seizure is not localized but lateralized to the right hemisphere during VEEG monitoring (The blue line was clinical seizure onset).

The neuropsychologist reported that the patient had retained her functional abilities and completed 12 years of education. Testing indicated that she struggled with complex visual memory and had slight impairments in basic attention span and cognitive flexibility. There were also indications of challenges in phonemic fluency and confrontation naming, which might be attributed to side effects from the medication. MRI of the brain showed negative results for lesions. PET showed hypometabolism in the right temporal and anterior insula cortex (Figure 1B). Genetic screening results indicate that there are no genetic traits associated with epilepsy.

### Seizure semiology and EEG findings from the VEEG monitoring

The scalp EEG recording was collected using 29 electrodes with the Neuvo 64-channel Amplifier & Profusion EEG 5 software (Compumedics Limited, Australia). Two types of seizure were observed during the monitoring:

Type 1 (awake): An aura of pressure in the hard palate and tingling sensations in the mouth. Then, drooling with copious saliva and inability to talk (she reported later that the accumulation of saliva made her unable to speak). She wiped her mouth with either her right or left hand. She was confused briefly but could speak clearly.
Type 2 (from sleep): Eyes open, she called her partner to warn of the seizure, then sat up, unable to talk (again, the accumulation of saliva made her unable to speak). She drooled copious saliva, which she wiped off with either her right or left hand. She looked confused and went back to sleep. On one occasion, her head and eyes were versed to the left, with left facial and upper limb clonic movement while wiping saliva.

**Figure 2:**
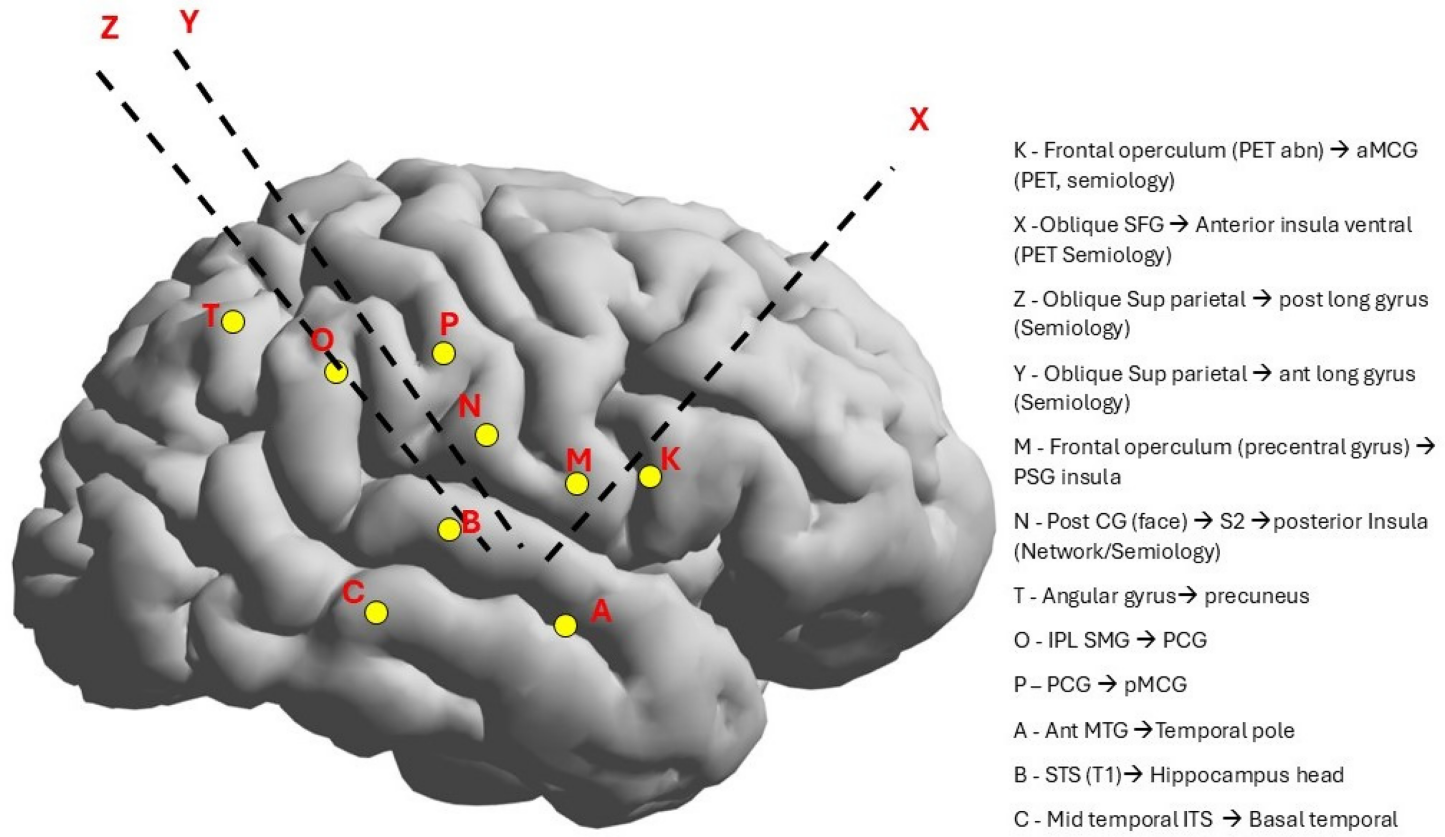
SEEG electrode implantation was based on the semiology and EEG findings, which were the right hemisphere and the insular-opercular region (the symptoms of hypersalivation and oral sensation).

The VEEG monitoring captured the two seizures. Seizure onset was not localized but lateralized to the right hemisphere, and interictal discharges were recorded in the right temporal region (Figure 1). After reviewing the case at a statewide epilepsy meeting, it was recommended that the patient undergo SEEG monitoring. The SEEG implantation plan was formulated based on the patient's seizure semiology, which included oral paresthesia and speech disturbances, predominantly impacting the insula-perisylvian region. The involvement of the mid and posterior insula is associated with paresthesia. Involvement of the posterior insula during a seizure may result in pain, hypersalivation, and clonic contractions of the left facial muscles, correlating with the right operculum regions. Ictal hypersalivation may also be present in patients exhibiting temporal lobe involvement (Figure 2). The patient then underwent a 10-day SEEG monitoring procedure. During this procedure, a neurosurgeon (DW), under the guidance of an epileptologist (AN), inserted 12 electrodes into the right insula-opercular regions, with good coverage of the Insula, opercular regions, somatosensory cortex, and temporal lobe, including the hippocampus.

The SEEG procedure involves implanting intracerebral 0.8 mm diameter electrodes (DIXI Medical, France) with 8–18 contact points (2 mm long and spaced 1.5 mm apart) using the Cosman-Roberts-Wells (CRW) stereotactic system, guided by pre-operative MRI scans. It utilizes a CRW frame (Integra LifeSciences Corp., USA) and drill guide, with intraoperative x-rays to determine electrode angles. For insulo-opercular epilepsy, an orthogonal approach samples both cortices with one lead, while an oblique approach primarily suits insular epilepsy (7). Electrode positions are confirmed with post-implantation brain CT scans. The SEEG data was collected using 12 electrodes with 180 contact points via the Neuvo 256-channel Amplifier and Profusion EEG 5 software (Compumedics Limited, Australia). During monitoring, 15 seizures were recorded. Anti-seizure medications were reduced, and hyperventilation was used as a provocation procedure. The irritative network surrounds the EZ and generates interictal epileptiform discharges (IEDs) without significant behavioral changes (8). This network includes regions adjacent to a lesion or epileptic focus exhibiting abnormal electrical activity. It does not generate seizures, it can facilitate their spread and influence clinical symptoms. In this case, IEDs were identified in the ventral anterior insula, the anterior long gyrus of the insula, the posterior short gyrus of the insula, the posterior long gyrus of the insula, the secondary somatosensory cortex, and the head of the hippocampus.

**Figure 3:**
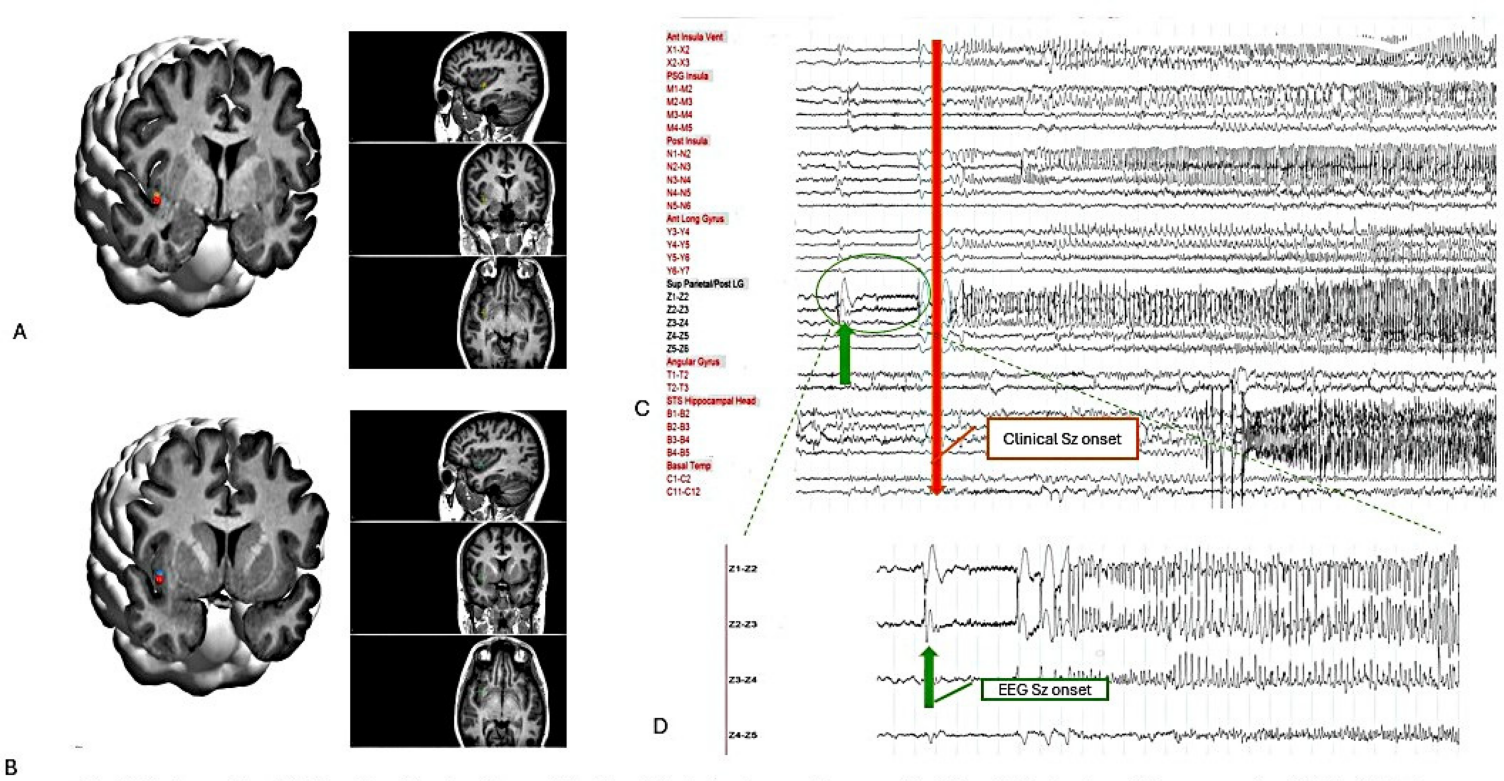
Postoperative 3-D imaging: the location of the Z and Y electrodes are shown on the T1-weighted volumetric preoperative MRI, which is fused with the postoperative CT using curry 9.0 software. **(A)** Z 3-4. **(B)** Y 3-4. **(C)** On bipolar montages, EEG seizure onset in Z 2-3 (green arrow) and clinical seizure onset (red arrow). **(D)** Zoom up EEG seizure onset-Z2-3 with LVFA.

The epileptogenic zone was identified in the right middle area of the posterior long gyrus of the insula (Z2-3), characterized by a direct current shift followed by low-voltage fast activity in Figure 3. Early involvement of the posterior and anterior ventral insula (Y3-4) exhibited low-voltage fast activity. The pattern of seizure propagation, which occurred with notable frequency, rapidly extended to the hippocampus. Although involvement of the opercular region was noted, the propagation pattern in this area was less consistent.

During the 8th and 9th days of SEEG monitoring, cortical stimulations were performed using Profusion EEG 6 software (Compumedics Limited, Australia) and the established stimulation protocol. For continuous low-frequency stimulation (1–9 Hz), electrical pulses of 1 millisecond duration were applied with a current intensity ranging from 1 to 5 mA. Each stimulation train lasted for 40–60 s. In contrast, for continuous high-frequency stimulation (30–60 Hz), electrical pulses of 1-millisecond duration were used with a current intensity ranging from 0.2 to 3 mm, and the duration of these stimulation trains was set to 7–8 s. When low-frequency stimulation at 6.0–9.0 Hz with 3.0 and 5.0 mA for 60 s was applied to regions Y 3-5, it resulted in after-discharges. High-frequency stimulation at 30 and 60 Hz with 3.0 mA for 5 s at Y 4-5 also produced after-discharges. Low-frequency stimulation at 6.0 Hz with 3.0 mA was applied to region Z 2-3 for 60 s, resulting in a non-stereotypical seizure, as the patient reported that the seizure onset felt different this time. When low-frequency stimulation at 9.0 Hz, with an intensity of 1.0–5.0 mA for 60 s, was applied to Z 2-3, it produced typical tingling or pressure sensations in the mouth's hard palate, which is considered a typical aura. Increasing the stimulation to 5.0 mA resulted in a stereotypical seizure characterized by the production of copious saliva.

**Figure 4:**
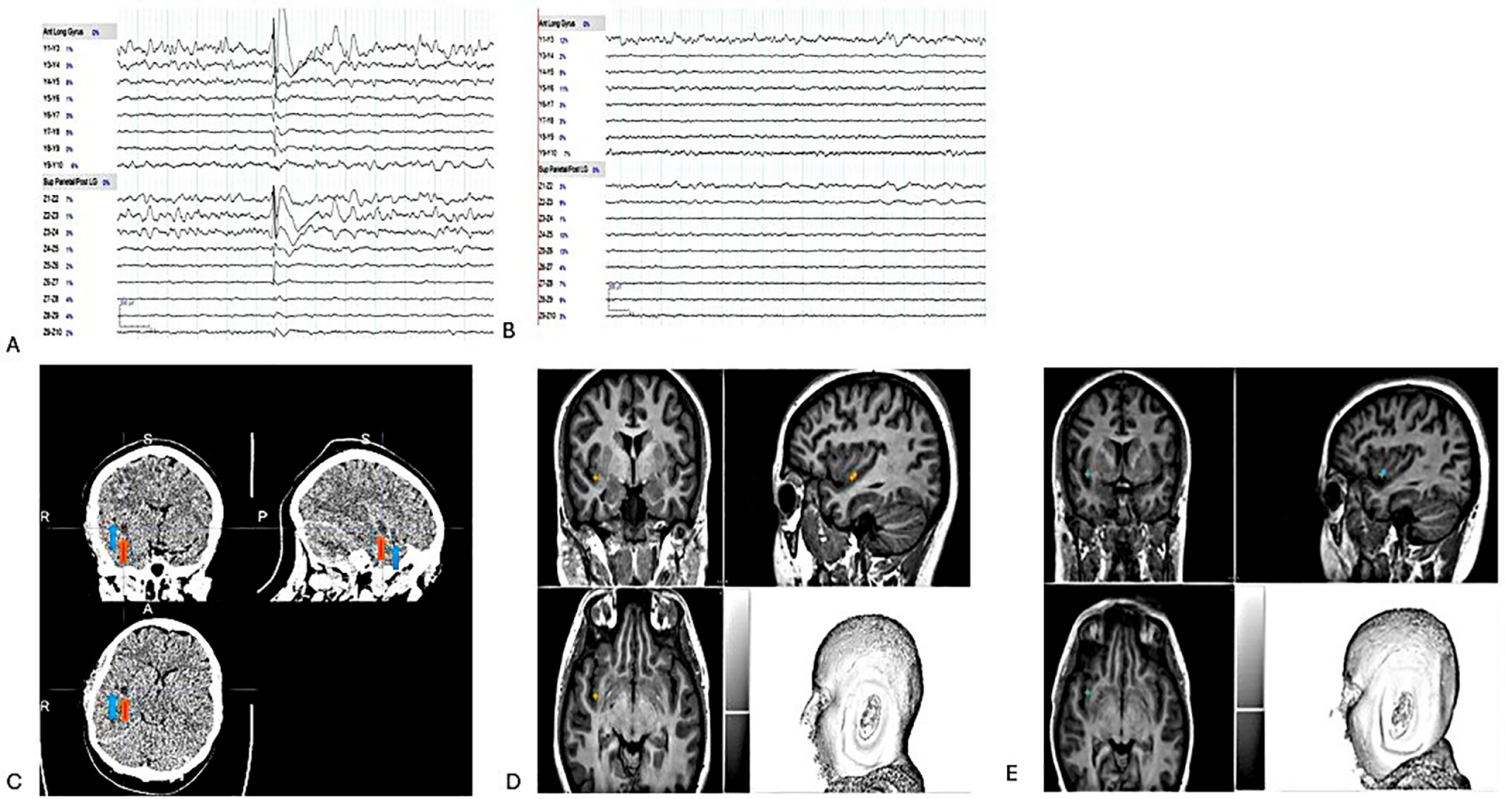
**(A)** Pre-RFTC -discharges in Y 3-4 & Z 2-4 on bipolar montage. **(B)** Post RFTC- flat lines in Y 3-4 & Z 3-4 on bipolar montage. **(C)** Z electrode, 3-4 (red arrow) & Y electrode, 3-4 (blue arrow) on CT scan post-RFTC. Two months post-T1 weight MRI results show no visible lesion. Therefore, Z and Y electrodes were placed to indicate their location, **(D)** Z 3-4. **(E)** Y 3-4.

Based on the results of the cortical stimulations and SEEG seizures, the SEEGg-RFTC procedure was performed by a neurosurgeon (DW) and an epileptologist (AN). It utilized a radio-frequency generator system (The Cosman RF system, Optimus Medical Limited, UK) that connected the Z 3-4 and Y 3-4 using current power gradually increased from 1.5W up to 8.32W within 60 s. These were the only contact points that were part of the early seizure network, not adjacent to vessels, and were deemed safe to use for RFTC. The delivered power determined the current intensity and voltage changes. These parameters could elevate the tissue temperature to 78°C–82°C and create an ovoid lesion with a long axis of approximately 7–10 mm between the two contacts. Due to the proximity of a large vein, RFTC could not be performed at the Z1-2. During the procedure, the patient expressed nervousness and reported, “Ewoo, Ewoo, oh my god, burning Yak! Yak! I heard a popping noise in my head”. SEEG monitoring was conducted to observe changes in epileptiform discharges (ED) in Z and Y electrodes before and after SEEGg-RFTC (Figures 4A,B).

The SEEGg-RFTC procedure was conducted without the use of anesthesia. A CT scan was performed three hours post-procedure, followed by an MRI scan two months later. The CT scan revealed the presence of gliotic scars (Figure 4C), whereas the MRI displayed no visible lesions (Figures 4D,E). It is important to note that visible lesions are usually seen on MRI after RFTC; however, no observable lesions were identified in this case. We have identified two potential explanations for the absence of a visible lesion on the MRI conducted two months post-treatment. The first explanation is “insufficient thermal injury”. If the temperature or duration of the RFTC procedure was inadequate, it may not have caused sufficient tissue damage to be detectable on the MRI. Incomplete coagulation can lead to a lesion that remains undetectable. The second explanation pertains to “subthreshold energy delivery”. If the energy delivered during the ablation was insufficient, the tissue may have had the capacity to recover without incurring permanent damage, resulting in an unobservable lesion. A 12-month post-lesion MRI is scheduled, highlighting its crucial role in all RF-TC cases. This procedure is essential for understanding the localization of the EZ and is vital for effective long-term management. The patient was discharged the following day. Post-RFTC, the patient exhibited complete seizure freedom (Engel class 1A) at all follow-up appointments at 24 h, three months, and six months. There were no recorded motor deficits or neurological symptoms associated with the SEEG implantations or the RFTC procedure. We evaluated the patient's quality of life (9), depression (10) and anxiety (11) status before and after the procedure, as shown in Supplementary Table S1. It's encouraging to observe a notable decrease in anxiety for this patient after RFTC.

## Discussion

The goal of epilepsy treatment is to enhance the quality of life by controlling seizures with minimal side effects. In this case study, seizures from the posterior insular cortex led to excessive salivation. Diagnosing and treating refractory epilepsy in this region is challenging due to its location at the intersection of multiple brain lobes (12). The symptoms of insular seizures often resemble those originating from the frontal, parietal, or temporal lobes. The insular cortex, also known as the “Island of Reil”, is located deep within the lateral sulcus of the brain. Reil initially described it in 1809 (6, 13). The insula cortex, hidden beneath the frontal, parietal, and temporal operculum, is highly vascular and connects to various brain areas, playing a role in memory, emotions, and sensory functions. Wilder & Penfield's work in the mid-20th century provided early insights into its role in the human insula (13, 14). They reported that the anterior part includes three short gyri—the anterior, middle, and posterior short gyrus—and an additional accessory gyrus on the ventral margin of the anterior part of the Insula. The posterior part has two long gyri—an anterior and a posterior long gyrus (9).

Isnard et al. examined the clinical features of insular lobe seizures by performing direct electric insular stimulations using depth electrodes (15). A review of 138 seizures in 50 patients found that five had discharges from the insula, highlighting the importance of accurate ictal onset localization for insular epilepsy. Symptoms included hypersalivation, facial clonus, and laryngeal constriction, with hypersalivation indicating discharge spread to the supra-sylvian operculum, often with dysarthria. Seizures featuring ictal hypersalivation originated in the posterior insula, with high-frequency spikes in the posterosuperior quadrant, identifying the insula and operculum as sources of salivary seizures. Solanki et al. conducted a study on the role of the insula in epilepsy. They found that the semiology of insular seizures varies based on specific subdivisions, and seizures can be diagnosed using SEEG (6). They also reported somatosensory manifestations were frequently observed, mainly when ictal discharges arise in the posterior insulo-opercular region. Mazzola et al. examined the use of intracranial electrodes to investigate stereotactic stimulations of the insular cortex and characterize the semiology of insular seizures (16). In this study, a total of 550 stimulations resulted in a clinical response rate of 82.2%. The most common responses were somatosensory, accounting for 61% (*n* = 335) of all responses. In contrast, visceral-sensitive sensations comprised 14.9% (*n* = 82), including salivation (*n* = 5). Notably, these responses originated from stimulation sites that were significantly located more anteriorly than other sites. It is important to note that the visceral-sensitive responses may have been underestimated due to the presence of the Sylvian vessels, which complicates electrode implantation in the anterior insula. According to these findings in the literature, the insula and operculum could be the areas most involved in ictal hypersalivation.

The SEEG technique, developed by Bancaud & Talairach, involves placing electrodes in the brain to record electrical activity to enable subsequent procedures (17). SEEG utilizing either oblique or orthogonal trajectories is considered a safer technique than intracranial strip or depth electrodes for insular cortex exploration due to the deep location and large numbers of adjacent blood vessels (18). However, given the insular cortex's location within the Sylvian fissure and its proximity to the middle cerebral artery (MCA), there is a risk of vascular injury (18). When implanting SEEG electrodes in the Insula, it is crucial to ensure adequate coverage as an insular seizure onset zone can be very focal (19). Alomar et al. found no intracerebral hemorrhages, and orthogonal and oblique trajectories were executed safely—insular electrodes aided in detecting insular epilepsy in 11.6% of the patients (20). Thorough coverage of the insulo-opercular region is imperative for differentiating insular from opercular seizure onset. This necessitates the utilization of lateral orthogonal trajectories through the frontoparietal and temporal operculum, as well as an oblique approach through the frontal or parietal cortices, to facilitate comprehensive sampling of the insula (19). Open surgical resection of the insula carries significant risks despite recent reports suggesting reasonable outcomes (6, 21).

SEEGg-RFTC offers a distinct advantage over surgery as it can use already implanted SEEG electrodes (4). In contrast, alternative surgical techniques such as laser ablation, focused ultrasound, and gamma-knife require separate procedures to determine the seizure onset zone (17). The SEEGg-RFTC procedure connects a radiofrequency generator to electrode contact points, utilizing current flow between two adjacent electrodes. This procedure occurs while the patient is awake, allowing for the monitoring of any undesirable neurological deficits. When conducted with care, RFTC is both safe and well-tolerated (17, 20, 21). Oliveira et al. conducted a study of the outcomes and safety of SEEGg-RFTC, focusing on patients with MRI-negative epilepsy. Nearly half (45.2%) of patients had a 50% reduction in seizure frequency, and 25.8% became seizure-free. Most (76%) showed no significant cognitive decline. SEEG-g-RFTC is a safe procedure leading to a good response in many cases of MRI-negative focal epilepsies.

There is often an approach to epilepsy surgery that relies on identifying radiologically visible lesions likely to cause epilepsy (22). MRI-negative patients tend to have a poorer prognosis, with only 20% achieving seizure freedom after extra-temporal surgery (23). However, non-lesional patients who underwent temporal lobe surgery can experience a significantly more favorable outcome, with nearly half shown to achieve seizure freedom two years postoperatively (23). Improvement in the early months following SEEGg-RFTC predicts a good outcome after conventional cortectomy in eligible surgery patients (24). Huang et al. reported that 36.7% of their MRI-negative epilepsy patients achieved seizure freedom with SEEGg-RFTC. Patients with the EZ in the insular lobe or with one focus on the limbic system were more likely to achieve seizure freedom (25).

Intracranial EEG recordings are used to locate the source of insular seizures using SEEG or depth electrode techniques. Low voltage fast-activity (LVFA is often seen at the onset of insula seizures (26). A 2023 review found that patients with LVFA at seizure onset had significantly higher rates of seizure-free outcomes (27). SEEGg-RFTC is a safe and effective treatment for drug-resistant focal epilepsy, especially in non-lesional neocortical epilepsy like periventricular nodular heterotopia (28). SEEGg-RFTC can support a favorable outcome after surgical or minimally invasive procedures if it improves seizure control (even temporarily) but fails to achieve seizure freedom (29). Mullatti et al. studied RFTC to improve seizure outcomes in insular epilepsy post-SEEG. RFTC can be performed after SEEG electrode removal or as a single MRI-guided procedure. In the study, 74% of patients had favorable outcomes (Engel class I or II), with 53% seizure-free and without permanent disability. Some required additional RFTC due to relapses. The research emphasized that volume-based RFTC can be curative for well-selected patients and that accurately identifying the epileptogenic zone is critical, with SEEG being the most effective method. The recommended coagulation volume is about 2 cm^3^ (21).

Jayapaul et al. studied outcomes in patients with refractory epilepsy undergoing resective or ablative surgery for insulo-opercular foci. They concluded that Radiofrequency Ablation (RFA) is safer than resection and emphasized the importance of analyzing auras, semiology, EEG, and imaging for better localization. Additionally, SEEG effectively identifies the epileptogenic zone in MRI-negative cases (30), leading to comparable outcomes to MRI-positive cases. Li et al. conducted a meta-analysis comparing the efficiency and safety of Magnetic Resonance-guided Laser Interstitial Thermal Therapy (MRgLiTT) and SEEGg-RFTC in epilepsy patients with focal cortical dysplasia (FCD). Both therapies showed low complication rates (17.1% overall, 2.5% long-term) with no significant differences (*P* = .17) (31).

The growing use of SEEG has led to an increase in the diagnosis of primary insular epilepsy (6). Accurately identifying the insular onset zone is imperative, as erroneous localization may lead to treatment failure (21). Historically, insular resection was not widely embraced due to challenges associated with accessibility. However, advancements in microsurgical techniques have substantially mitigated risks. Presently, permanent morbidity rates are approximately 8%, with no documented mortalities (32). The surgical outcomes for non-lesional insular epilepsy are favorable in 60%–90% of patients. The commonest morbidities of insular resection are hemiparesis and speech disturbances. Motor deficits may result from direct damage to the pyramidal tract or vascular compromise (6). Minimally invasive surgery techniques are currently available.

Regarding surgical resection, Lehe et al. reported that insular lesionectomy achieved a satisfactory seizure outcome (32). Kerezoudis reviewed the outcomes of insular epilepsy surgery (33) and showed that the most common surgical approach was the trans-sylvian method (60%), with the anterior insular region being the most frequently removed (21%). After a median follow-up of 29 months, 60% of pediatric and 69% of adult patients were seizure-free. However, 43% of cases experienced neurological deficits, with 10% permanent. Obaid et al. conducted a review on resective surgery for insula epilepsy (34), finding that 66.7% of patients were seizure-free post-surgery. Post-operative neurological complications occurred in 42.5% of patients, with motor deficits most common (29.9%). Removing the frontal operculum during surgery can lead to motor deficits, and dominant-hemisphere surgeries may result in temporary language deficits. However, surgery may be necessary if the frontal operculum is part of the EZ. In most cases, removing part or all of the insular cortex in patients with drug-resistant epilepsy does not cause significant permanent neuropsychological impairment (35). SEEGg-RFTC is an excellent treatment option for non-lesional insular epilepsy. In the event of treatment failure leading to induced lesions in the insular cortex, patients can confidently proceed with surgical interventions.

## Conclusion

Epilepsy is recognized as a network disease, and diagnosing ictal hypersalivation involving the Insula can be intricate due to its potential to mimic other conditions and multifaceted functionalities. Understanding insular seizure symptoms is crucial for an accurate diagnosis. Technological advancements have significantly improved the safety of insular cortex sampling. While scalp EEG findings are generally uninformative for the deep-265seated Insula, SEEG is an effective method for investigation. SEEGg-RFTC offers an alternative to surgical intervention and may lead to periods of seizure freedom. Although associated with favorable outcomes, surgical intervention carries inherent risks, particularly in dominant insula resections. A comprehensive understanding of insular seizure symptoms is essential for identifying insular epilepsy, especially when MRI does not reveal anomalies.

## Data Availability

The original contributions presented in the study are included in the article/Supplementary Material, further inquiries can be directed to the corresponding author.
