# Supplementary material for: Case Report: Ictal hypersalivation: a stereoelectroencephalography exploration
Source: Front Surg. 2025 Feb 26;12:1535408. doi: 10.3389/fsurg.2025.1535408 (PMC11897517; doi:10.3389/fsurg.2025.1535408)
Supplement: Supplementary file 1 [file Table1.docx]

Table 1: Assessments of quality of life and mood: QOLIE-10-P: Quality of Life in Epilepsy Inventory-10-questions; NDDI-E: Neurological Disorders Depression Inventory in Epilepsy; GAD-7: General Anxiety Disorder-7 questions.

| Assessment | Pre RFTC | Three months Post RFTC |
| --- | --- | --- |
| **QOLIE-10** |  |  |
| Part A – *How much of the time during the past 4 weeks:* |  |  |
| 1. Did you have a lot of energy? | 4 (Some of the time) | 5 (A little of the time) |
| 1. Have you felt downhearted and low? | 4 (Some of the time) | 1. (A little of the time) |
| *How much of the time during the past 4 weeks have your epilepsy or antiepileptic drugs caused trouble with:* |  |  |
| 1. Driving (or other transportation) | 4 (Only a little) | 5 (Not at all) |
| *During the past 4 weeks:* |  |  |
| 1. How much do your work limitations bother you? | 3 (Sometimes) | 3 (Sometimes) |
| 1. How much do your social limitations bother you? | 4 (Only a little) | 3 (Sometimes) |
| 1. How much do your memory difficulties bother you? | 5 (Not at all) | 4 (Only a little) |
| 1. How much do physical effects of antiepileptic drugs bother you? | 3 (Sometimes) | 2 (A lot) |
| 1. How much do psychological effects of antiepileptic drugs bother you? | 3 (Sometimes) | 4 (Only a little) |
| 1. How afraid are you of having a seizure during the next 4 weeks? | 1 (Very afraid) | 2 (Somewhat afraid) |
| 1. How has your QUALITY OF LIFE been during the past 4 weeks? | 3 (Good & bad about equal) | 2 (Pretty good) |
| Part B – *Reviewing all the questions answered in Part A, consider the overall impact of these problems on your quality of life in the past 4 weeks* |  |  |
| 1. How much does the state of your epilepsy-related quality of life distress you overall? | 3 (Moderately) | 3 (Moderately) |
| Part C - Please indicate the areas related to your epilepsy that are most IMPORTANT to you NOW. Number the following topics from ‘1’ to ‘7’ with ‘1’ corresponding to the most important topic and ‘7’ to the least important one. | 1. Quality of life  2. Mental activity  3. Energy  4. Emotion  5. Worry about fits  6. Medication effects  7. Daily activity | 1. Quality of life  2. Mental activity  3. Energy  4. Emotion  5. Daily activity  6. Medication effects  7. Worry about fits |
| **NDDI-E** |  |  |
| *Please input the answer that best describes you in the past weeks, including today. How much of the time:* |  |  |
| 1. Everything is a struggle | 3 (Sometimes) | 3 (Sometimes) |
| 1. Nothing I do is right | 2 (Rarely) | 2 (Rarely) |
| 1. Feel guilty | 3 (Sometimes) | 2 (Rarely) |
| 1. I’d be better off dead | 1 (Never) | 1 (Never) |
| 1. Frustrated | 4 (Always) | 4 (Always) |
| 1. Difficulty finding pleasure | 3 (Sometimes) | 3 (Sometimes) |
| **GAD-7** |  |  |
| *Over the past 2 weeks, how often have you been bothered by the following problems?* |  |  |
| 1. Feeling nervous, anxious or on edge | 2 (More than half the days) | 1 (Several days) |
| 1. Not being able to stop or control worrying | 2 (More than half the days) | 0 (Not at all) |
| 1. Worrying too much about different things | 2 (More than half the days) | 0 (Not at all) |
| 1. Trouble relaxing | 1 (Several days) | 1 (Several days) |
| 1. Being so restless that it is hard to sit still | 2 (More than half the days) | 1 (Several days) |
| 1. Becoming easily annoyed or irritable | 2 (More than half the days) | 2 (More than half the days) |
| 1. Feeling afraid, as if something awful might happen | 1 (Several days) | 0 (Not at all) |
